# Navigating memory problems and mild cognitive impairment in later life: A qualitative secondary data analysis

**DOI:** 10.1177/14713012251335380

**Published:** 2025-04-17

**Authors:** Christine Carter, Penny Rapaport, Claudia Cooper, Henry Llewellyn, Michaela Poppe, Marina Palomo, Hassan Mansour, Paul Higgs

**Affiliations:** Centre for Psychiatry and Mental Health, Wolfson Institute of Population Health, 4617 Queen Mary University of London, UK; Division of Psychiatry, 4919 University College London, UK; Centre for Psychiatry and Mental Health, Wolfson Institute of Population Health, 4617 Queen Mary University of London, UK; East London NHS Foundation Trust, UK; Division of Psychiatry, 4919 University College London, UK; Central North West London NHS Foundation Trust, UK; Research Department of Clinical, Educational and Health Psychology, 4919 UCL, UK; Centre for Psychiatry and Mental Health, Wolfson Institute of Population Health, 4617 Queen Mary University of London, UK

**Keywords:** identity, liminality, relationships, age, subjective, active ageing intervention, memory concerns

## Abstract

Many people experience memory concerns as they get older, which can produce uncertainty and confusion. Some seek help for memory concerns and receive diagnoses of Mild Cognitive Impairment or Subjective Cognitive Decline, while most do not. We aimed to explore the subjective experiences of people with cognitive concerns, and how memory impairment is understood in the context of ageing. We undertook a secondary data analysis of 18 in-depth semi-structured interviews with people aged 60+ living with memory concerns, 9 of whom had sought professional help. We thematically analysed the data, identifying three themes. 1. *Situating Memory Concerns*: people are required to situate their experiences within contradictory discourses: medicalised, diagnostic categories, and reassurances that symptoms are normal, part of ageing and distinct from dementia. 2. *Affirming self in the face of memory loss:* individuals refer to social roles in families, work, and associated activities to reaffirm identity. For some, activities were a vital anchor of identity, resulting in negative self-comparison to their past self if abilities declined. 3*. Maintaining* identity *and Relationships;* labelling memory concerns helped relatives seeking external validation of symptoms, but not the person experiencing them, for whom it brought no additional support. Consequently, some participants concealed memory concerns. We concluded that the subjective experiences of people with memory concerns were characterised by confusion, and diagnostic labels compounded this rather than offering reassurance, bringing tension to some relationships. Activities were often perceived as an outward sign of continuing identity, leading to distress when ability to continue them declined. We discuss these issues in relation to broader societal issues around age and ageing.

## What is known about this topic

• Evidence suggests that Mild Cognitive Impairment remains a contested term, characterised by a lack of clarity.
• Individuals will paradoxically experience not having dementia whilst still being at risk of developing dementia, the trajectory for which is unclear.
• We theorise that people with Mild Cognitive Impairment and memory difficulties, who do not have dementia, experience being in a liminal position.

## What this paper adds

• Highlights the importance of understanding the subjective experiences of memory concerns and Mild Cognitive Impairment.
• Illustrates the effect of receiving or not receiving a diagnosis of Mild Cognitive Impairment on an individual’s identity and relationships with others.
• Reveals the complexity surrounding how people navigate age and memory difficulties, extending the ideas of liminality and including the third and fourth age.

## Introduction

Many people worry about their memory, especially as they age (Cooper et al., 2011; Maxfield et al., 2024). This is understandable as age is the major risk factor for dementia (NICE, 2024) (National Institute for Health and Care Excellence). The estimated number of people living with dementia worldwide is projected to increase to 115 million by 2050, so it is a major focus of global health prevention strategies (Prince et al., 2013).

Focus on preventing and diagnosing dementia at an early point has increased the numbers of people living with heightened awareness of cognitive difficulties, even though in most cases these are not symptoms of a dementia illness. Individuals who have cognitive symptoms are at high risk of developing dementia. These symptoms are often termed, or labelled by health professionals, as Subjective Cognitive Decline subjective impairment in the absence of objective impairment, and Mild Cognitive Impairment objective cognitive impairment exceeding what would be expected for one’s age, without significant daily impairment (Gauthier et al., 2006; Jessen et al., 2014). Previous research suggests that, when these diagnostic criteria are applied to general populations subjective cognitive decline and mild cognitive impairment affect, respectively, 50% and 20% of adults over the age of 65 (Lopez et al., 2007; Stewart, 2012).However, accurate rates are difficult to establish, Gillis et al. (2019) systematic review concluded that a heterogeneity in mild cognitive impairment incidence estimates persists across age specific estimates. In the UK, older adults presenting to primary care with these conditions have a nearly 50% chance of receiving a diagnosis of dementia within three years (Hallam et al., 2022). Most individuals meeting the criteria for mild cognitive impairment are undiagnosed, so these labels are ascribed to a minority who seek early help for memory concerns.

While a diagnoses of mild cognitive impairment may for some be empowering and lead to adopting preventative lifestyle measures that may reduce risk (Bird et al., 2021), their utility has been questioned. They can generate uncertainty and confusion for those who, paradoxically are seeking validation that they do not have dementia (Gerstenecker & Mast, 2015; Pickersgill, 2014; Schweda et al., 2018). Labelling symptoms of memory or cognitive loss can make them feel like an illness, even though they are often part of normal human experience (Leibing, 2018a). Dhedhi suggested that a focus on early identification of cognitive concerns might lead to over-diagnosis of dementia and stigma (Dhedhi et al., 2014).

The emphasis on early dementia diagnosis has been regarded as an aspect of the medicalisation of normal forgetfulness in ageing and the blurring of “normal to pathological” distinction (Pickersgill, 2014). Those given the mild cognitive impairment “label” are distinguished from those having dementia while simultaneously being informed that they are also at risk of developing it (Swallow, 2020). Health professionals use mild cognitive impairment narratively both to resolve uncertainty as well as to avoid it (Swallow, 2020). Gerstenecker & Mast (2015) describe an over-reliance on the measurement of memory and a minimisation of subjective experiences regarding the nature of “normal ageing” when making a diagnosis of mild cognitive impairment. The medicalisation of mild cognitive impairment and memory impairment often fails to account for subjective experiences of the condition, including fear and a desire for individuals to distance themselves from indicators of dementia (Beard & Neary, 2013; Dooley et al., 2020; Leibing, 2018b; Poppe et al., 2020).

Medicalised discourses of memory impairment and the role of a diagnosis of mild cognitive impairment have become important in contextualising contemporary ageing. In this paper, we consider memory concerns and mild cognitive impairment against a backdrop of sociological thinking around ageing. We acknowledge Bury’s work (Bury, 1982) on biographical disruption, discuss notions of liminality, and utilise the theoretical concept of distinction (Libert et al., 2020). We draw upon conceptual models of later life as being bifurcated into what has been described as the third and fourth ages (Gilleard & Higgs, 2010) to help think about and discuss participants’ subjective experiences. This approach considers later life as marked by two differing cultural contexts: firstly, the third age, being marked by social engagement and individual agency. This is contrasted with the fourth age which articulates an old age marked by growing cognitive, physical, and social losses. In this formulation, the third age can be regarded as a positive cultural field encompassing choice and consumerism but which in doing so shapes a negative “social imaginary” of the fourth age, which according to Gilleard and Higgs projects a representation of a “feared old age” of dependency, limited choice and marginal agency (24). We address the need for research to include subjective accounts of the lived experience of mild cognitive impairment and memory impairment in the context of these cultural tropes. We also sought to explore how participants navigated the challenges that individual memory concerns and mild cognitive impairment bring to their expectations of later life, including those around personal identity.

## Methods

### Procedure and setting

This paper reports a secondary analysis of data collected for a qualitative study, designed to inform development and co-production of the APPLE-Tree (active prevention in people at risk of dementia: lifestyle, bEhaviour change and technology to REducE cognitive and functional decline) intervention. The APPLE-tree programme is a lifestyle and wellbeing intervention, delivered through workshops with people aged 60+ who have memory concerns to which no diagnostic label has been ascribed, as well as those diagnosed with mild cognitive impairment (Cooper et al., 2020). The purpose of the primary qualitative study was to explore how the APPLE-tree programme should be designed and delivered (Poppe et al., 2020, 2022). The original study involved semi-structured, face-to-face qualitative interviews with 45 participants including National Health Service (NHS) staff, third sector staff, people living with memory concerns or mild cognitive impairment and family carers between July and September 2019. The methods of the primary study and topic guides are described in full by Poppe (Poppe et al., 2020). Here, we focus on interviews with people living with memory concerns, some of whom had been diagnosed with mild cognitive impairment. This differs from the original research focus as we explore the subjective experiences of memory difficulties including how individuals navigate challenges related to this and ageing.

Participants were recruited purposively for mild cognitive impairment or memory concerns from NHS memory services, Improving Access to Psychological Therapies (IAPT) services for common mental health problems and third sector organisations across London, South England, and North-West England. This included people aged 60 and above, who scored 50+ (screening negative for dementia) on the QuickMCI Screen (O’Caoimh et al., 2012). We only included participants scoring 62+ who answered “yes” to at least two of the following three questions, designed to detect subjective cognitive decline: “*Has your memory deteriorated in the last 5 years? Or has a friend or family member noticed it deteriorating?; Is your memory persistently bad? Or has a friend or family member noticed it being persistently bad?; Are you concerned about this? Or are others around you concerned about this?*” We adapted this approach from published measures, full details of APPLE-Tree inclusion criteria are provided in the study protocol (Cooper et al., 2020).

Interviews explored how people with memory concerns are motivated and supported to make targeted behavioural changes, and the potential barriers and facilitators to participating in, and delivering an active dementia prevention programme.

### Rationale for secondary data analysis

Requirements for using secondary data state that researchers “authentically account for the nature of the original data set and have knowledge of the original data set” (Thorne, 2013). Four authors of this paper undertook the interviews, which enhanced familiarisation with the data. Questions contained within the interview schedule focused on experiences of memory problems in individuals’ lives. The nature of the data set aligned well with our research aim which is distinct from the primary analysis.

### Analysis

We used reflexive thematic analysis to analyse the interviews (Braun & Clarke, 2021) in line with our specific research aim (above). Where a relative or partner accompanied the interviewee and occasionally took part in the interview, we focused upon the interview data from the person with memory difficulties and or mild cognitive impairment, unless where relevant to the analysis and emerging themes. One author (CC) was immersed in the transcript data re-reading transcripts and listened to a sample of five interview recordings. We adopted a reflexive approach, using a diary to record reflections during the analysis, and then systematically coded all transcripts into meaningful fragments, creating initial codes. Codes were discussed with other authors and then developed into clusters of codes and then themes. Themes were discussed through a reflexive process with the results written and re-written as part of the analysis.

## Findings

### Sample description

The sample contained 18 participants, including 4 women and 14 men. Five people had a formal diagnosis of mild cognitive impairment. Nine people described themselves as having memory concerns and had sought professional advice but had not received a diagnosis, and four described having memory concerns but had not sought professional advice. Table 1 outlines the sample characteristics.

**Table 1.** Sociodemographic and cognitive characteristics of the sample.

Type of memory concern	Number	Gender	Age range
Mild cognitive impairment diagnosis	5	2 female 3 male	61–92 72–78
Memory concerns with professional advice	9	2 female 7 male	61–68 62–83
Memory concerns with no professional advice	4	4 male	73–87

Participants who had sought professional advice or received a diagnosis of mild cognitive impairment described repeated contacts with services and had single to multiple tests. Some were unclear about what testing they had received, or indeed if any had taken place.

## Qualitative analysis

Three key themes were generated, giving insight into the individual experiences of memory concerns. Quotes from interviews are used to illustrate the complexity of this experience. Table 2 provides the theme names.

**Table 2.** Theme names.

Situating memory concern
Affirming self in the face of memory loss
Maintaining identity and relationships

### Theme 1: Situating memory concerns

This theme refers to how memory concerns were situated in terms of people’s experiences, commonly this was through distinction from dementia. For those who received a diagnosis this was experienced as a relief but also a threat, as participants were aware of the future risk of dementia and felt ill informed by services about how to manage this risk. This involved individuals developing understanding and knowledge of mild cognitive impairment, whilst navigating the language surrounding it.

Most individuals had not received a formal diagnosis of mild cognitive impairment. Health service professionals and participants appeared to use a range of terms to describe memory concerns. Frequently this meant that participants had to navigate varied and often contradictory discourses. The resultant anxiety is captured in the extract below between the researcher and husband and wife.

The main thing I would say is just more information, a lot more information. We don’t get enough. You were in there, like, ten minutes, weren’t you? They didn’t really tell you much, and then you were out. That was it. One leaflet (husband of person with Mild Cognitive Impairment (MCI)

One leaflet. (female participant 2 diagnosed with MCI)

It might turn into dementia, and then you’re thinking all this; checking online (husband)

I know. They don’t tell you really a lot online, do they? They tell you bits of things, don’t they? (female participant 2)

Well, no one knows. Also, when you go online, it can be scary stuff as well (interviewer)

It’s all different information (husband).

Regardless of whether a diagnosis was made, participants described common experiences of how information about causes, prognosis and possible remedies for memory loss was delivered, received, and understood. Some individuals with a diagnosis of mild cognitive impairment appeared to find the diagnosis reassuring as the extract below illustrates.

Now that I have been diagnosed, I feel okay (male participant 2 diagnosed with MCI)

And before that? Before you had the diagnosis? (interviewer)

Where I was always thinking, what is wrong, what is wrong with me, and then this is very mild compared to what I could be looking at in ten years (male participant 2)

The above quote reflects participant’s difficulty in predicting future progression of memory impairment following a diagnosis. The relief at the absence of dementia may be tempered by the presence of the unknown.

Expectations around age were significant factors in clinical consultations and diagnosis affecting how individuals situated and made sense of impairment. The exchange below was framed within the context of normal ageing and physical health with phrases such as “nothing to worry about”. The intended reassurance or confirmation that an individual’s memory concern is “just” related to age often left several unanswered questions. This 77-year-old man was asked about an exchange with his GP (General practitioner) when he sought help regarding his memory difficulties, and it was unclear to him whether he had received a diagnosis.

Yes, he (GP) understood, but he said you haven’t got a problem. It’s an age-related thing. (male participant 15 with memory concerns)

Okay, so it’s age rather than anything more serious? (Interviewer)

No, nothing medical. (male participant 15)

Did he give you any kind of information about what you can do? (Interviewer)

No, No. and I don’t think it was taken that seriously. I don’t think he saw it as a problem. I saw it as a problem, but I don’t think he did. (male participant 15)

In attempting to distance memory problems from a medical explanation, the GP gives an explanation that it is normal and “age related”. The significance of “age” in a consultation provides evidence of not having “other” problems, that is dementia. Alternatively, locating the responsibility on individuals to self-monitor memory function and notice deterioration was also evident, as the following exchange illustrates.

They [the GP] basically said she had the mild cognitive impairment, and just gave her a little leaflet about doing exercises and stuff. (husband of person with MCI)

But they didn’t even tell me really what it was or anything. (female participant 1 diagnosed with MCI)

No, she [GP] didn’t explain (husband)

She [GP] said if it gets worse come back (female participant 1)

Although empowering for some, judging “if” or when things become worse involves self-monitoring and an ability to seek help at an appropriate time, all of which may become harder as memory deteriorates.

### Theme 2: Affirming self in the face of memory loss

People described reaffirming their self-identity, agency, as well as their control over their own lives through participation in various activities. Advice about health promotion might help to re-frame memory impairment from being a marker of deterioration to it becoming a potentially treatable condition, raising positive expectations for the future for participants. Individuals utilised existing social and physical activities in order to minimise the effects of impairment, often in the face of adversity. They frequently drew upon previous roles and activities to affirm who they are, as shown in the extract below:

Because I was very busy as a businessman before I retired and I was very conscious that I had to have the plan in place to do things. The only plan I didn’t have in place was to keep my memory, so there we go, that’s where it is (male participant 7)

As illustrated here, individuals were confronted by the reality that previous roles and experience may not help to manage memory impairment, comparing their current self negatively to their past self.

Individuals frequently sought to define their identity in terms of what they did, who they are, and who they were in context of their memory concerns. This is illustrated below by a female participant discussing past activities:

I am trying, yes, because I am that sort of person. I am interested. Obviously, as a university graduate. I am a book person. An Arts graduate. I am a word person, yes. (female participant 5)

Avoiding dementia through retaining one’s identity was a key motivation and keeping physically active facilitated individuals to re-position themselves and reinforce their sense of self. One male participant talked about how he viewed engaging in and maintaining such activities as “stopping him disappearing”.

Well, I think being physically active and getting fit, must increase the blood flow through the system and stops you from… What’s the word? Disappearing, or you know, just sitting down and not doing anything. (male participant 10)

For some there was a sense that activities anchored their identity, leading some of them to negatively compare their current self with their past self if their abilities had appeared to decline. Additionally, there was also a pressure not to “give in” to memory failings with individuals using phrases such as “*carrying on”* and *“just doing it”* when they were describing their memory concerns, this included expressions of anxiety.

Although individuals did not directly equate memory impairment with well-being, on closer analysis, expressions of anxiety and fear were often voiced. Lack of control and knowledge about dementia produced feelings of uncertainty and anxiety as described below.

I do worry about my memory because it’s getting worse, I know it’s getting worse. And I don’t want to get dementia. That’s the last thing I want to get because I’ve seen people with it, and I know what it’s like. (female participant 2)

Physical and mental health concerns combine and can affect well-being through an individual’s ability and motivation to take part in activities such as exercise. This is exemplified below after this female participant was asked about food and diet as part of the consultation study.

Well, if you had a proper diet and you had proper exercise, and you had something to get up for in the mornings, you know, I’ve got nothing to get up for really. When I get up in the morning, it’s the same thing every day, nothing ever changes. And I think it gets you down, and I suffer from anxiety as well. (female participant 3)

There are wider issues around mental health and well-being demonstrated here. For example, socio-economic status along with co-morbidities may limit an older person’s ability to adopt health and lifestyle changes, affecting their mood and general mental wellbeing (Baiyewu et al., 2020). Discussions around health and lifestyle which may be motivating may conversely also trigger feelings of anxiety if the person feels they should be doing something which they are not.

### Theme 3: Maintaining identity and relationships

This theme refers to the impact of memory impairment upon relationships with family and friends. Individuals described feeling harassed by family members who they felt were irritated and exasperated by their behaviour. Getting a diagnosis of mild cognitive impairment may be regarded as resolution by relatives, seeking external validation however, without resultant support it can feel exposing for individuals. This is highlighted by a male participant who was speaking about a situation where his wife accompanied him to the GP.

What’s annoying me slightly about it, because we went to the doctor’s about it and she insisted on coming…. She said, “I wanted you to know what you’re really like” (male participant 10)

The impact of memory impairment had a profound effect on relationships with others. Avoiding difficulties being reflected back on to the participant is challenging when memory impairments are overt. This can create stress, with the consequence that individuals can avoid further contact. This is captured by the participant below when asked who else had noticed her memory impairment.

People that I know that I’ve forgotten to meet them at a particular place. And they’re angry or I’ve forgotten to call them when I should have or... that kind of thing…I prefer not to go, I prefer not to socialise actually. I prefer to stay at home and not to go out somewhere. (male participant 9)

How individuals relate to others is to some extent affected by how other people relate to them and their memory difficulties as well as how signs of their memory impairment are reflected to them.

The experience of memory impairment generally involves strategies to manage or conceal deficits in memory. These can range from those which were regarded as more “acceptable” to those which required active concealment. Concealing memory concerns and impairment was key to participating in everyday life. A female participant decided not to tell family and friends about her diagnosis and described in detail a situation of not remembering where she had parked her car,

“whenever I would park to do an exercise class or visit a friend, my car, I would not be able to see it. Even remember the colour or where I parked it. So, then I have the embarrassment of pretending from a mile away that I am bleeping my car. I changed my car to get over this”. (female participant 2)

Changing her car to one where the lights flashed when pressing the key remotely, allowed her to locate the car after being out with friends or visiting family and conceal her difficulties. When asked at what point she might tell her family she replied she would *“bat it back for a long a possible”* and *“just get on with it”*. This demonstrates the range of strategies adopted from everyday problem solving to complex decisions in order to retain autonomy, control and agency over a participant’s life. It also highlights the pressure some individuals experience to cover-up memory difficulties for as long as possible. The pressure to maintain relationships was also apparent, with memory concerns impacting a couples’ relationships. The extract below is from a married couple who were being interviewed together, where the husband has memory impairment.

Is there anything we haven’t already covered? (interviewer)

Personally, I’m not the person, am I? But we think … if you could talk about these conversational difficulties and perhaps give us a clue as to what to do. Probably the answer is you can’t. But I did talk to somebody here last week and he was really nice and said, the partners can contact .....but we’ve never had a chance, have we, to talk to somebody about how [unclear] impacts around the relationships.”(wife of male participant 1)

Understanding the impact of memory impairment on an individual within a family and on a family system is essential in order to start to fully understand their lived experience. This includes recognising the potential burden of knowing when and how to return to memory services if things change.

## Discussion

In this study we analysed data from interviews with people with memory concerns or mild cognitive impairment exploring how participants made sense of their cognitive impairment in the context of ageing. We identified three themes which are situated within a context of expectations surrounding age and normal ageing. Knowledge and information received from clinicians was described by participants as being limited and unclear, producing confusion. Within the public arena, normal ageing increasingly equates to “successful ageing” (Esmaeili, 2020; Rowe & Kahn, 1997), with its implied expectations to undertake healthy interventions to slow cognitive deterioration, following confirmation of memory impairment, with or without a diagnosis (Jones & Higgs, 2010). We found that the affirmation of personal identity is sought and navigated in multiple ways, including negotiating strategies to avoid and conceal memory impairment. These strategies serve to distance individuals from the “fear” of developing dementia. Relationships are complicated by memory impairment and appear to affirm and support identity.

## The utility of a diagnosis

Individuals find an mild cognitive impairment diagnosis difficult to understand. This makes the utility of a formal diagnosis uncertain (Dooley et al., 2020; Gerstenecker & Mast, 2015; Schweda et al., 2018). However, we found individuals embraced a “diagnosis” of “age related problems” as evidence of not having “other” problems, that is dementia. Paradoxically, this diagnosis also indicated a risk of developing dementia. Society’s increasing focus on a “cure” for dementia, part fuelled by the media and projected warnings of demographic increases in prevalence, has been termed the “Alzheimerisation of ageing” (Adelman, 1995; Gilleard & Higgs, 2014). Contexts of dementia are resetting the co-ordinates of what old age means in contemporary society (Higgs & Gilleard, 2017; Higgs & Jones, 2009). Alongside increased life expectancy and qualitative improvements in older age in contemporary societies, these have influenced the expectations individuals hold about mild cognitive impairment and cognitive concerns.

## Mild cognitive impairment as a chronic illness

The experience of memory impairment is perhaps difficult to situate in standard accounts of physical chronic illness such as Burys’s theory of “biographical disruption” (Bury, 1982). Bury describes chronic illness as a major disruptive event disabling one’s relationships and ability to get resources. He explores how people engage in processes of resource mobilisation to “normalise” the face of disruption, all of which has resonance with memory impairment and mild cognitive impairment. However, mild cognitive impairment is unlike other chronic health conditions such as arthritis which carry a level of permanence connected to a progressive course of anticipated decline. Any possible stability is countered by the “need” to prevent dementia and the liminality surrounding the condition. Poppe et al. (2020) refers to liminality as an experience of “lostness” among people with memory concerns who find themselves in a transitional state “betwixt-and-between” the normal age-related decline in memory and the pathological notion of disease. Mild cognitive impairment is contextualised as being different from dementia as well as from normal ageing, the lack of a clear categorical boundary is experienced by those with memory loss as being both blurring and confusing.

## Identity

Our themes around identity and relationships suggest that reaffirmation of self-identity can be a strategy employed to keep memory problems “at bay”. McGee’s study of the virtues and strengths of a people living with early-stage dementia describes the need for health care providers to provide encouragement and validation of personal identities (McGee et al., 2023). Despite reassurances that memory problems are not dementia, they can strongly affect relationships, impacting on self-identity. Relationships can challenge how people respond to memory concerns, forcing people to see how they are in other people’s eyes. Retaining strategies to conceal memory impairment, mitigate this and maintain identity, and relationships require “extra cognitive work” (Chaudoir et al., 2013). Our study revealed examples of concealment, we found that the experience of memory concerns poses challenges to relationships which the diagnosis of mild cognitive impairment does not seem to allay, at least for the person experiencing memory concerns.

## Liminality

Birt previously described liminality in relation to dementia as a progressive disease with marked landmarks and transitions (Birt et al., 2017). Mild cognitive impairment does not readily fit this characterisation, although considered part of a pre-clinical transition phase towards dementia this is ambiguous. Not all people with mild cognitive impairment go on to develop dementia and a diagnosis is not crucial in an individual’s desire to avoid developing dementia.

Most people in our study had not received a formal diagnosis of mild cognitive impairment. Their experiences suggest a broader understanding of liminality, rather than the static or transitional state of illness progression previously considered. Pervasive liminality considers how individuals encounter discrete experiences which overlap and fluctuate over time, rather than in-between experiences associated with a liminal space (Bruce et al., 2014), which is supported by our findings.

## Sociological approaches

Experiences of forgetfulness and age-related memory loss shift toward being re-defined as examples of cognitive ill health and impairment. Higgs and Gilleard (2020) adopt a reflexive approach concentrating on the interplay between the culture of the third age and the social imaginary of the fourth age. The contrast between the third and fourth age provides a way of thinking about the role of mild cognitive impairment and memory concern as components of a metaphorical “event horizon” (Gilleard & Higgs, 2010) between the pull of a negative representations of the fourth age and the agency associated with the third. Dementia alongside frailty, abjection, and the need for care project a “feared” old age irrespective of attempts to de-stigmatise it. Paradoxically the mild cognitive impairment label also distinguishes people as not having “too much” dementia and therefore gives people agency to affirm their location as being primarily in the third age through engaging in, “active cognitive health promotion” (Libert et al., 2020).

Examples from our findings support the view that individuals are expected to navigate the management of their own memory with an explicit assumption they have the capacity to recognise when their memory is declining, and to respond to such changes through adaptive strategies (Berg et al., 2013). This relates to the discourses of both active ageing and responsibilisation in the arena of health and ageing, (Varey et al., 2021). Existing research describes experiences of liminality for people with mild cognitive impairment and memory impairment as conferring a sense of responsibility (Birt et al., 2017; Poppe et al., 2020) and the idea that dementia has “modifiable” risks which can be incorporated into everyday life. Libert et al. (2020) have used the concept of technologies of distinction in his work to study brain training and healthy ageing discourses. They posit how this has been used to illustrate how individuals demonstrate that they are actively resisting cognitive decline and as such positioning themselves from others in a third age culture which distances a feared fourth (Libert et al., 2020).

## Policy and practice implications

Our findings illustrate the complex experiences of people with memory concerns, with or without mild cognitive impairment diagnoses, highlighting important points for public health.

Memory loss can disrupt “normal” ageing but mild cognitive impairment places individuals in a liminal position in being less biographically disruptive but with an inference of individual responsibility for memory deterioration. This may lead to an individual being asked to accept the idea of memory loss at an earlier stage than might be necessary which needs consideration within public health.

Active ageing has expanded notions of active cognitive ageing along with the corresponding normative expectations and assumptions for the older population (Libert et al., 2020). A mild cognitive impairment diagnosis becomes an impetus to act and engage in active ageing strategies to prevent cognitive deterioration. Mild cognitive impairment is a term used to galvanize people, a call to action to stave off a feared future with dementia. This potentially places a moral burden on individuals to act to prevent or forestall dementia, which warrants further consideration.

Diagnostic status appears of less importance than the impact of memory difficulties and mild cognitive impairment upon identity. Creation of strategies to mask and live with the effects of memory impairment and age “normally” or “successfully” require consideration. Understanding how identity is affirmed, constructed, and maintained is an important part of acknowledging the subjective nature of memory impairments. Policy makers must understand how the experiences of mild cognitive impairment and memory impairment are layered with broader social and individual responses to age and ageing, encompassing negotiating relationships and identity.

## Strengths and limitations

The interviews did not directly address our aims, and this is a limitation of secondary data analysis. However, the area of study and method of analysis aligned with the primary data set. Hinds et al. (1997) identifies different approaches to justify using secondary data analysis, one of which is appropriate for our study where the focus is on a different unit of analysis from that of the parent study. Sharing data is a legitimate way to use the consultation data effectively and lead to the generation of new knowledge whilst addressing a sensitive area of research (Long-Sutehall et al., 2011). We were interested to note that more of our participants identified as male than female, and curious as to whether some responses to memory concerns might differ with gender, potentially an interesting topic for future study. Our findings provided insight into the complexity of participants’ experience of memory concerns and mild cognitive impairment diagnosis, illustrating the need for further direct research in this area.

## Summary

We sought to understand subjective experiences of people living with memory concerns and those diagnosed with mild cognitive impairment through analysing interview transcripts from the Apple-tree study’s consultation phase. Our themes describe experiences which are tempered by societal responses to cognitive decline which include assumptions about health, age, and personal responsibility. Individuals with mild cognitive impairment and memory impairment feel the need to create meaning, exercise control and avoid cognitive deterioration. Active ageing through a health and lifestyle intervention to prevent cognitive deterioration may enable this. However, this engagement is complicated and highlighted through our themes around affirmation of identity, expectations and age, relationships with others, and the navigation of unclear information.

## Data Availability

The qualitative dataset is accessible from the authors on receipt of a reasonable request.*
